# Genetic Diversity of a Wild *Actinidia arguta* Population in Changbai Mountain Determined by Simple Sequence Repeat Markers

**DOI:** 10.3390/cimb47030207

**Published:** 2025-03-19

**Authors:** Xiaowu Sun, Guangli Shi, Songze Li, Jun Ai, Dan Sun, Zhenxing Wang, Peijin Ni, Zhendong Zhang, Shuaiming Chen, Zelong Du, Xiang Li, Fan Zhang

**Affiliations:** College of Horticulture, Jilin Agricultural University, Changchun 130118, China

**Keywords:** *Actinidia arguta*, wild populations, genetic diversity, simple sequence repeat (SSR), variation

## Abstract

With the decrease in the number of natural populations of *Actinidia arguta*, there is an urgent need to collect *A. arguta* germplasm resources and explore their genetic diversity for better management and protection. In this study, 31 simple sequence repeat (SSR) markers were used to identify 148 wild *A. arguta* germplasms from six natural populations in Changbai Mountain, China, and the genetic diversity of their leaf quality traits was subsequently evaluated. SSR analysis revealed rich genetic diversity among different individuals and within populations of *A. arguta*. Molecular variance analysis determined that the genetic diversity of wild *A. arguta* mainly came from within the populations (95% variance component ratio), while only a small part originated from among populations (5% variance component ratio). Abundant genetic variations were observed in the leaf quality traits of the different *A. arguta* resources with a high genetic diversity index (0.13–1.23). Leaf quality trait clustering and the unweighted pair group method with arithmetic average (UPGMA) clustering analysis showed similar classification results. Population structure analysis divided 148 individuals into three subgroups. Our results indicate that the populations of *A. arguta* in Changbai Mountain have large genetic variation and high genetic diversity. This study broadens the genetic basis of the *A. arguta* breeding germplasm.

## 1. Introduction

*Actinidia arguta* is a perennial deciduous woody vine belonging to Actinidia. It is mainly distributed in China, the Korean Peninsula, most of Japan, and the Russian Far East. It grows in humid areas (e.g., mountains and streams) at an altitude of 25–3600 m [1,2]. The fruit of *A. arguta* is small, thin, smooth, unique in flavor, and has a special aromatic odor [3]. It is rich in flavonoids, polysaccharides, alkaloids, saponins, and other active ingredients [4]. *A. arguta* has strong cold resistance (minimum tolerance to low temperatures of −30 °C) and a short growth period (150 d) [5], making it an attractive plant to grow across the world (including China, the United States, New Zealand, Italy, Belgium, Poland, the Netherlands, and France) [6]. Compared to *A. chinensis* and *A. deliciosa*, *A. arguta* has a higher resistance to bacterial canker [7].

Genetic diversity (changes in genes and genotypes) represents the heritable variation within and between biological populations [8]. As a basic component of biodiversity, genetic diversity is important for the long-term survival of any species [9]. Generally, genetic diversity refers to the degree of genetic variation in phenotypic traits, cell level, and molecular level within or between individuals. The extensive investigation and genetic diversity evaluation of germplasm resources are important in the basic research of genetics and genomics, germplasm conservation, and breeding [10]. The investigation of biological traits is the most intuitive index to indicate the genetic diversity of plants, including the investigation of phenotypic traits, such as branches, leaves, and fruits. Many scholars have evaluated and studied the genetic diversity of phenotypic traits in species populations such as *Carya illinoinensis* [11], *Malus pumila* [12], *Diospyros kaki* [13], *Pinus koraiensis* [14], and *Arabidopsis thaliana* [15]. At present, research on the genetic diversity of wild populations of *A. arguta* is limited. Previous studies have only focused on the variation in agronomic traits and ploidies of *A. arguta* populations in the Daba Mountains and Qinling Mountains in Shaanxi province. Scholars have also analyzed the genetic diversity of five *A. arguta* populations distributed from northeast to southwest China [16,17,18]. Although the main distribution area of wild *A. arguta* is in the Changbai Mountain area, reports on the genetic diversity of the population in this region are lacking. As a result, the understanding of the natural population of *A. arguta* in the Changbai Mountain area is limited, preventing the systematic clarification of its genetic variation patterns and genetic diversity levels.

Simple sequence repeat (SSR) is also called microsatellite DNA, and its core unit is a repetitive DNA sequence composed of 1–6 bp motifs. SSR belongs to the second generation of molecular marker technology; the core technology is Polymerase Chain Reaction (PCR) amplification and gel electrophoresis. SSR has the advantages of rich polymorphism, co-inheritance, multi-allelic, simple operation, and convenient detection, and it is often used in molecular markers [19]. At present, SSR molecular marker technology has been applied to evaluate the genetic diversity of a variety of plant populations, such as *Ziziphus mauritiana* [20], *Mangifera indica* [21], *Carya illinoensis* [22], and *Litchi chinensis* [23].

This study identified 148 *A. arguta* resources from six geographical populations in Changbai Mountain by SSR markers. Following this, the genetic variation, differentiation among and within populations, and genetic diversity of their leaf quality traits were analyzed and evaluated. The purpose of this study is to explore the genetic diversity information of wild *A. arguta* natural populations in the Changbai Mountain area. The results help to explore the origin and evolution process of polyploidy in *A. arguta* and to divide the genetic relationship of wild resources so as to provide a theoretical basis for the breeding, division of genetic relationships, and classification of new varieties of *A. arguta*. At the same time, the results are conducive to future work on the genetic improvement, forest resource management, and germplasm protection of *A. arguta*.

## 2. Materials and Methods

### 2.1. Plant Materials

A total of 148 germplasm resources were collected from six natural wild populations distributed in the Changbai Mountain area (Table 1). The resource populations from Jilin City, Yanbian Prefecture, Baishan City, and Tonghua City in Jilin Province and Huanren County and Dandong City in Liaoning Province were denoted as JL, YB, BS, TH, HR, and DD, respectively.

From September to October 2022, mature lignification branches with a thickness of 0.5–1.0 cm were collected and transported to the laboratory of the College of Horticulture, Jilin Agricultural University, China. The collected dormant branches were divided into two parts and cut into small segments of about 40 cm in length. After one month of low-temperature sand storage to meet the cold demand, they were taken out, washed in water, and cultured at room temperature. When the branches of the hydroponics germinated and expanded at room temperature, the young leaves were picked for DNA extraction. The other part of the collected branches remained in the cold-proof pit for low-temperature sand storage until the second year for subsequent experiments. In the spring of 2023, 148 germplasm resources were grafted and stored in the germplasm resource nursery of *A. arguta*. The mature leaves in the middle of the branches were selected to investigate the genetic diversity of leaf phenotypic quality traits.

### 2.2. Genetic Diversity Analysis of Leaf Quality Traits

**Figure 1:**
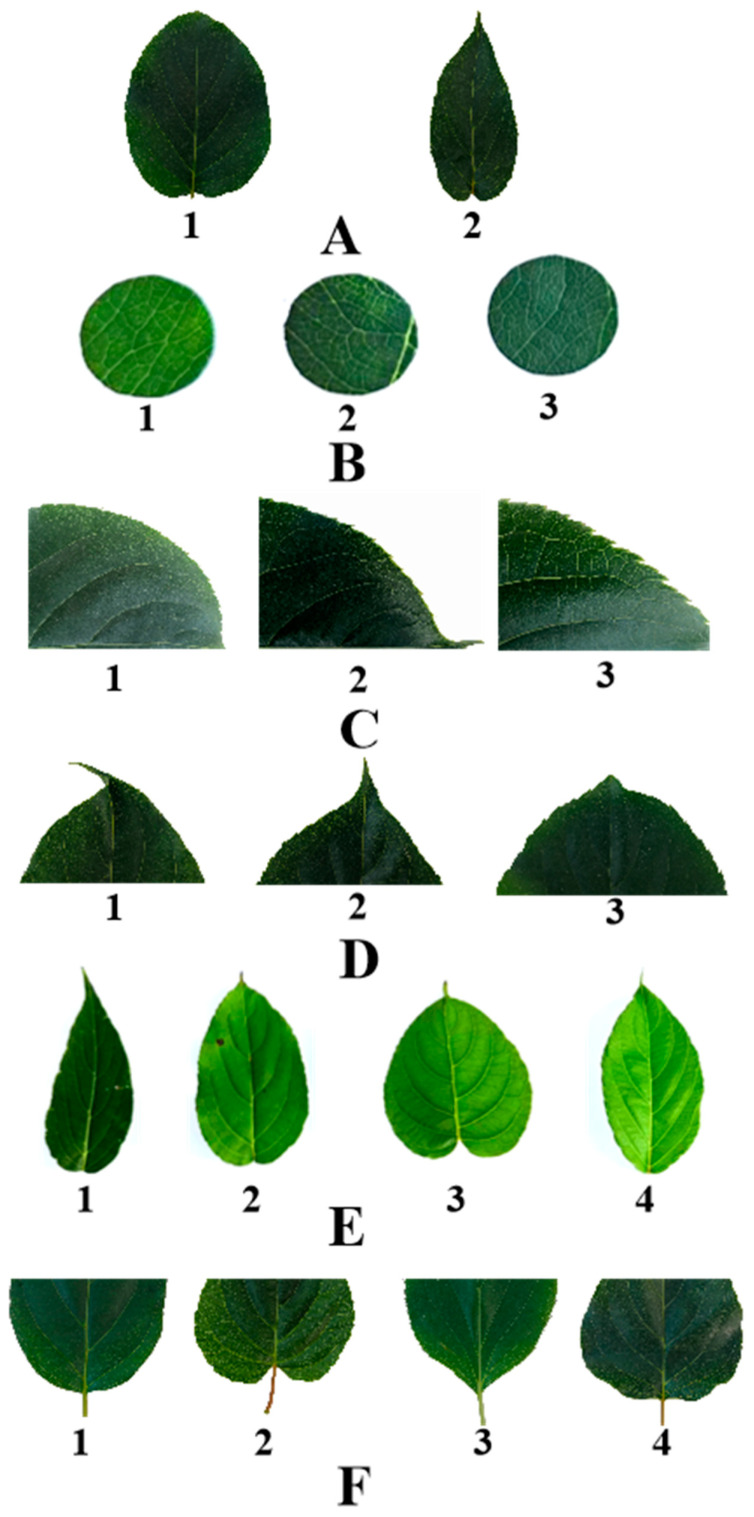
Grading standard of leaf phenotypic traits of *A. arguta*. (**A**) represent leaf margin: 1 is sawtooth, 2 is Undulation; (**B**) represent leaf color: 1 is Aqua, 2 is green, 3 is Bottle green; (**C**) represent leaf margin serration: 1 is Thin single serrations, 2 is Coarse single sawtooth, 3 is Two-out complex serration; (**D**) represent leaf tip shape: 1 is tail tip, 2 is Sharp, 3 is Taper; (**E**) represent leaf shape: 1 is Ovoid form, 2 is Broad oval, 3 is heart shape, 4 is oval; and (**F**) represent leaf base shape: 1 is Roundness, 2 is heart shape, 3 is wedge, 4 is Lopped section.

According to the description by Ai et al. [24] (Table 2, Figure 1), the leaf quality traits of the resources collected from the six populations of *A. arguta* in Changbai Mountain were investigated. In the fruit growth period, the adult leaves in the middle of the new shoots were employed to evaluate the following leaf-related traits: leaf shape, leaf tip shape, leaf margin, leaf margin serration, leaf base shape, and leaf color.

### 2.3. DNA Extraction and Quality Assay

DNA extraction followed the method described by Hanania et al. [25], with minor modifications. Approximately 0.1 g of young leaves was quickly ground in liquid nitrogen, lysed with CTAB (65 °C), extracted twice with chloroform, precipitated with pre-cooled isopropanol (−20 °C) for about 0.5 h, and finally washed with 75% ethanol 2–3 times. After drying, an appropriate amount of Tris-EDTA buffer (TE) was added to dissolve the DNA, and the sample was stored at −20 °C. The DNA quality was detected by 1.5% agarose gel electrophoresis, and the qualified samples were diluted to 10–20 ng/μL.

### 2.4. SSR Primer Screening and PCR Amplification

The 140 pairs of primers used in this experiment were derived from previous transcriptome data of the research group. The total volume of the amplification reaction system was 16 μL, including 8 μL of 2 × Taq PCR StarMix, 1 μL of template DNA, 0.8 μL of SSR forward and reverse primers, and 16 μL of dd H_2_O. The PCR amplification procedure was as follows: pre-denaturation at 94 °C for 4 min, denaturation at 94 °C for 1 min, annealing for 1 min (annealing temperature varied with the primer), extension at 72 °C for 1 min, 30 cycles, final extension at 72 °C for 7 min, and preservation at 4 °C. After PCR amplification, the amplification products were separated into 5% polyacrylamide gel electrophoresis and stained with silver.

### 2.5. Data Analysis

The distribution frequency of each qualitative trait in the population was calculated and evaluated using the Shannon–Weaver diversity index (H′), which was determined as follows:

H′ = −∑ Piln(Pi),

where Pi is the percentage of the number of materials in the ith category of a trait to the total number of copies and ln is the natural logarithm [26]. The leaf phenotypic traits were evaluated, and the results were statistically analyzed in Excel 2016 (Microsoft Corp. Washington, USA). SPSS 23.0 (IBM) was used for cluster analysis. The default Euclidean distance was employed as the cluster statistic, and cluster analysis was performed by intergroup connection to construct a tree diagram [27].

The PCR amplification results were classified as follows: characteristic target bands were assigned as ‘1’; non-target bands were assigned as ‘0’; and blurred and unidentifiable bands were recorded as missing. Excel 2016(Washington, USA) was used to input and establish the 0/1 matrix according to the presence or absence of bands. The original matrix was transformed into the format required for each software program using Dataformarter [28]. POPGEN 32(CA) was employed to calculate the observed number of alleles (*Na*), the effective number of alleles (*Ne*), Shannon’s information index (*I*), the observed heterozygosity (Ho), and the expected heterozygosity (He) of six populations and 31 pairs of primers [29]. Based on the amplification results of the 31 pairs of SSR primers, the genetic distance of 148 germplasms was calculated by NTSYS 2.0e.(Applied Biostatistics Inc., USA) The unweighted pair group method with the arithmetic average (UPGMA) method was used for clustering, and the clustering effect diagram was plotted in MEGA6.05(Pennsylvania, USA).

GenAlEx 6.5(AU) was used to calculate the genetic differentiation coefficient (*Fst*) and gene flow (*Nm*), which was determined as follows [30]:

*Nm* = 0.25(1 − *Fst*)/*Fst*;

then, analysis of molecular variance (AMOVA) was performed using GenAlEx 6.5 to compare the degree of genetic differentiation between and within the six wild *A. arguta* populations [31]. The genetic distance and genetic similarity coefficient between populations were calculated using the Similarity module in NTSYS 2.0e, and cluster analysis was subsequently performed between populations [32]. The population structure of 148 individuals was analyzed by Structure2.3.4 (Palo Alto, CA, USA) software [33].

## 3. Results

### 3.1. Genetic Diversity Analysis of Leaf Quality Traits

The leaf quality traits of the six populations of the *A. arguta* germplasm resources are presented in Supplementary Table S1. The genetic diversity index (H′) of the six phenotypic quality traits of leaves ranged from 0.13 to 1.23 (Table 3). The genetic diversity index of leaf phenotypic traits was ranked as leaf shape > leaf base shape > leaf color > leaf tip shape > leaf margin serration > leaf margin. The high genetic diversity index indicated that there were great differences in the leaf phenotypic quality traits of the wild *A. arguta* resources and that the genetic variation types were abundant.

**Figure 2:**
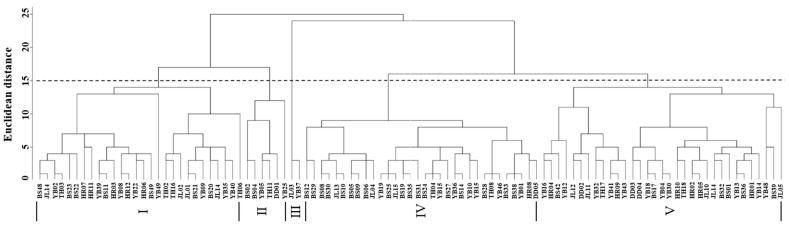
Cluster analysis of the leaf phenotypic traits of *A. arguta* germplasms by intergroup connection. JL, YB, BS, TH, HR, and DD represent the populations of Jilin City, Yanbian City, Baishan City, and Tonghua City in Jilin Province and Huanren County and Dandong City in Liaoning Province, I–V represent five groups respectively, respectively.

**Figure 3:**
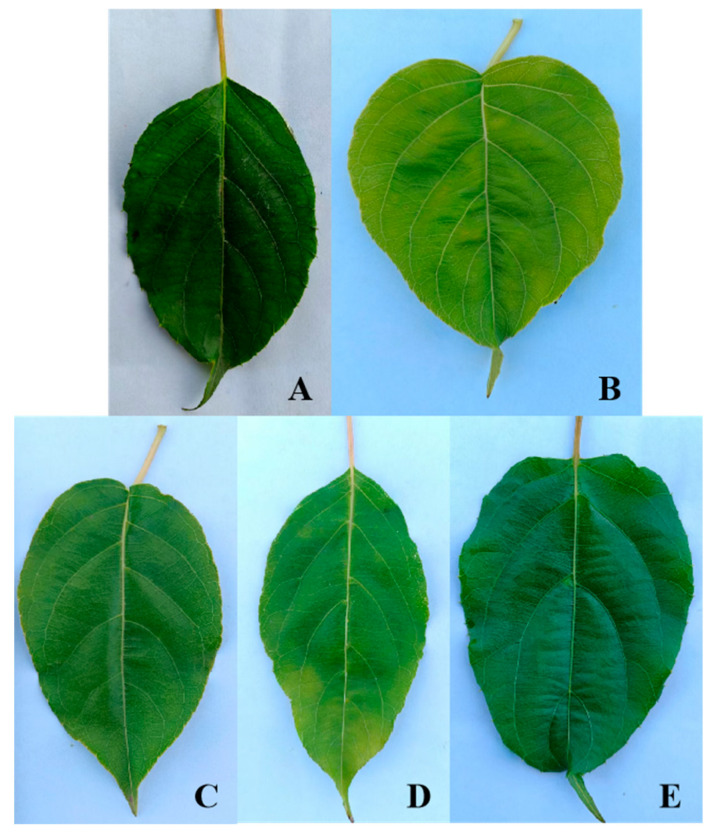
Representative leaf phenotypic combination of traits of 5 groups. Groups (**A**–**E**) represent Groups I, II, III, IV, and V, respectively.

Cluster analysis of the leaf phenotypic quality traits of the *A. arguta* germplasm resources was performed by the group connection method (Figure 2). At the Euclidean distance of 15, the germplasms could be divided into five groups. The representative leaf phenotypic traits of each group are shown in Figure 3. The first group included 28 germplasms, such as BS48, JL14, and YB02. In this group, the leaf shape was elliptical, the leaf tip shape was a tail tip, and the leaf base was mostly wedge-shaped. The second group included six germplasms, such as BS02, BS04, and YB05, which were mainly characterized by heart-shaped leaves and leaf bases. The third group included just two germplasms, JL03 and YB37, which were mainly characterized by an oval leaf shape, an acuminate leaf tip shape, and a green leaf color. The fourth group included 32 germplasms, such as BS12, BS29, and BS08. In this group, the leaf shape was mostly oval, the tip shape was tail tip, and the serration was fine single serration. The fifth group included 33 germplasms, such as YB16, HR04, and BS42, which were mainly characterized by an oval leaf shape, fine single-leaf margin serrations, and a dark green leaf color.

**Figure 4:**
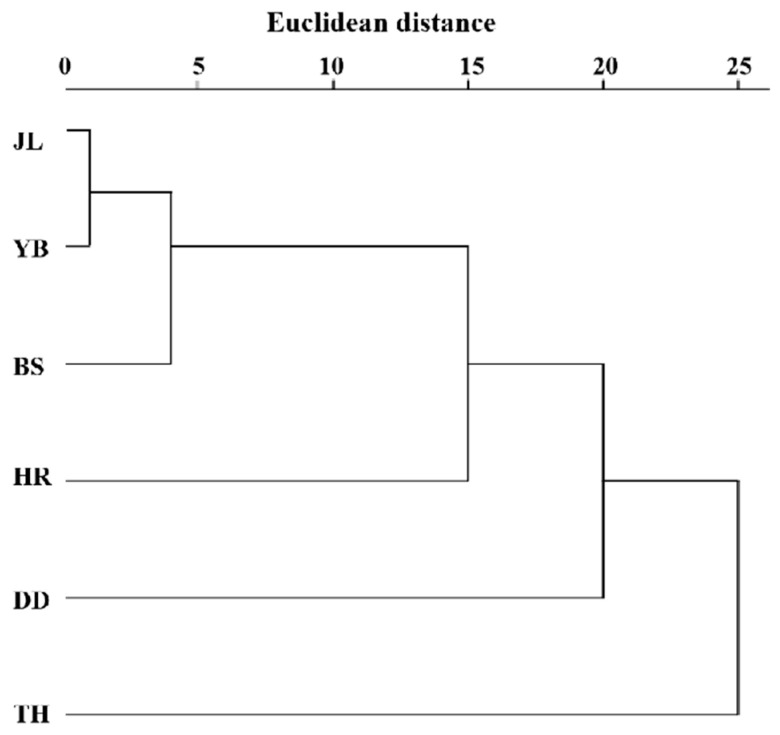
Cluster analysis of the leaf phenotypic traits of 6 *A. arguta* populations. JL, YB, BS, TH, HR, and DD represent the populations of Jilin City, Yanbian City, Baishan City, and Tonghua City in Jilin Province and Huanren County and Dandong City in Liaoning Province, respectively.

The clustering results of the leaf phenotypic quality traits of the six populations of *A. arguta* (Figure 4) were generally closely related to the geographical distance distribution, except for the TH population. The six populations were divided into two categories at the Euclidean distance of 24, with TH in one category and the other five populations in the other category. Three groups were determined at the Euclidean distance of 20, with TH in one category, DD in one category, and the other four populations in the remaining category. At the Euclidean distance of 15, four categories were determined, with TH in one category, DD in one category, HR in one category, and the other three populations in the remaining category.

### 3.2. Polymorphism Analysis of SSR Markers

The information on 31 pairs of SSR primers is in Supplementary Table S2. Six populations of *A. arguta* were analyzed by 31 pairs of SSR primers. The average percentage of polymorphic bands was 94.70%. The average number of observed alleles (*Na*) and effective alleles (*Ne*) were 1.932 and 1.707, respectively. Shannon’s information index *I* ranged from 0.521 to 0.604. The average observed heterozygosity (Ho) and expected heterozygosity (He) were 0.409 and 0.394, respectively (Table 4). Shannon’s information index *I* indicated that the genetic diversity in each population of *A. arguta* was abundant. The observed heterozygosity was greater than the expected heterozygosity, revealing that there was excessive heterozygosity in the population of *A. arguta*. Excessive heterozygosity suggests excessive gene flow in each *A. arguta* population, resulting in an increase in genetic diversity.

### 3.3. Analysis of the Genetic Diversity of A. arguta Germplasm

**Figure 5:**
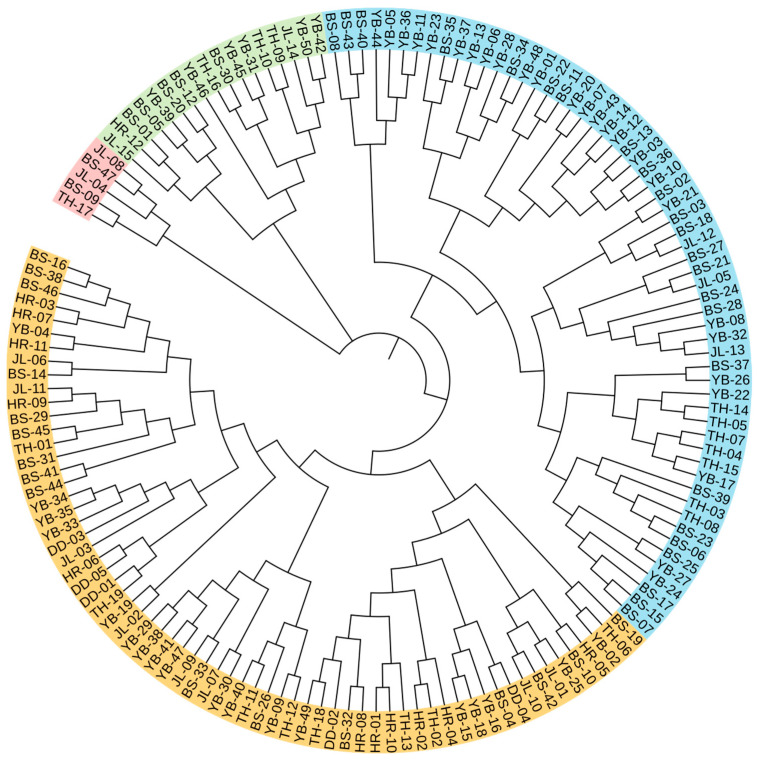
Individual cluster analysis of 148 *A. arguta* germplasms. Red, green, blue, and orange represent groups I, II, III, and IV. JL, YB, BS, TH, HR, and DD represent the populations of Jilin City, Yanbian City, Baishan City, and Tonghua City in Jilin Province and Huanren County and Dandong City in Liaoning Province, respectively.

The individual cluster analysis results are shown in Figure 5. At the genetic similarity coefficient of 0.82, the germplasm was divided into four groups. Group I contained five germplasm resources, including two from JL, two from BS, and one from TH. Group II contained seventeen germplasm resources, including two from JL, six from YB, five from BS, one from HR, and three from TH. Group III contained sixty germplasm resources, including three from JL, twenty-six from YB, twenty-four from BS, and seven from TH. Group IV contained sixty-six germplasm resources, including eight from JL, eighteen from YB, sixteen from BS, eleven from HR, five from DD, and eight from TH.

**Figure 6:**
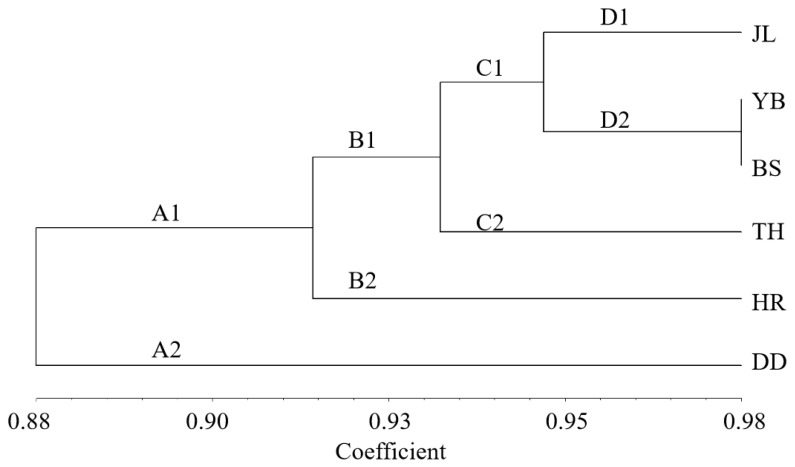
Cluster analysis of 6 *A. arguta* populations. JL, YB, BS, TH, HR, and DD represent the populations of Jilin City, Yanbian City, Baishan City, and Tonghua City in Jilin Province and Huanren County and Dandong City in Liaoning Province, respectively.

The unweighted pair group method with arithmetic average (UPGMA) cluster analysis based on Nei’s genetic distance of the six populations (Figure 6) shows the genetic relationship of the six populations. The populations were divided into two subclades. One subclade (A1) included five populations (JL, YB, BS, TH, and HR), and the other subclade (A2) contained only one population (DD). This indicates that YB and BS had the closest genetic relationship. Moreover, JL and TH had a close genetic relationship, while the genetic relationship between HR and DD was distant. Based on the geographical location of the six populations, we found that the results of the UPGMA cluster analysis were closely related to the relative geographical location of the six populations. More specifically, the closer the relative geographical location, the closer the genetic relationship between the populations.

### 3.4. Analysis of the Population Structure of the Tested Materials

**Figure 7:**
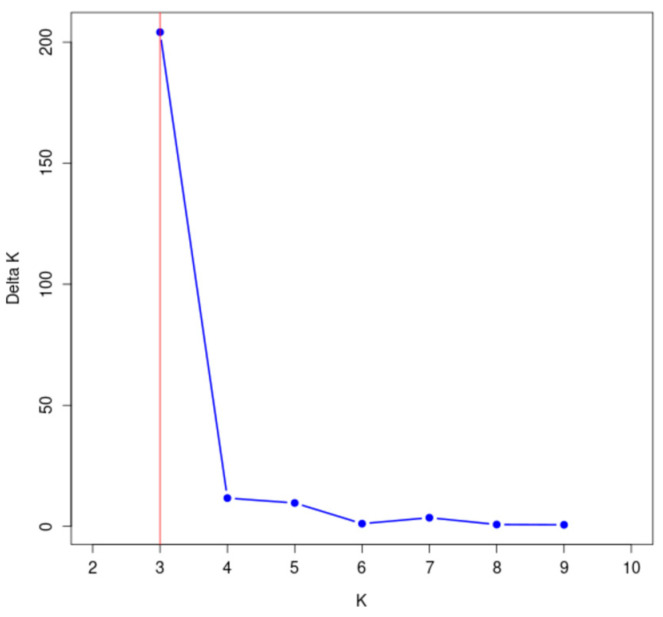
Change diagram of the delta K value corresponding to the K value.

**Figure 8:**
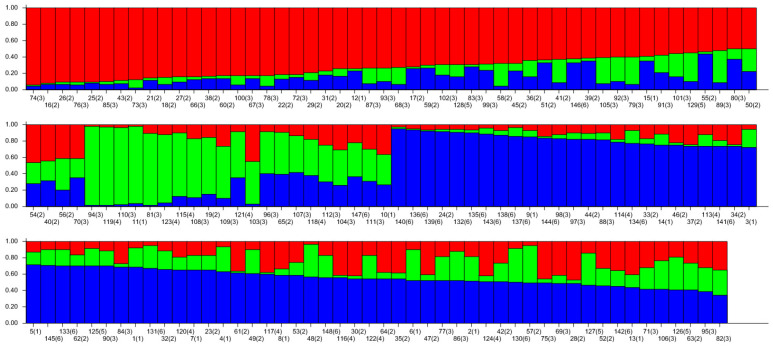
Population structure of 145 wild *A. arguta* based on SSR markers. The values 1, 2, 3, 4, 5, and 6 represent the populations of JL, YB, BS, HR, DD, and TH; 1–15 from JL, 16–57 from YB, 58–112 from BS, 113–124 from HR, 125–129 from DD, and 130–148 from TH, with 3 colors representing 3 distinct ancestral groupsi.

The genetic structure of 148 wild *A. arguta* germplasms was analyzed by Structure 2.3.4 software based on the genotypic data obtained from 31 pairs of primers. The highest peak in < DELTA > K was at K = 3 (Figure 7), and thus, the tested materials were divided into three subgroups. According to the value of ΔK, the distribution map of the population structure was constructed (Figure 8). According to the value of Q, the wild *A. arguta* germplasm materials could be classified into corresponding subgroups. A total of 148 germplasms were divided into three subgroups. Subgroup I contained fifty-four germplasms, including two from Jilin City, twenty-six from Yanbian Prefecture, twenty-three from Baishan City, two from Dandong City, and one from Tonghua City. Subgroup II contained twenty-one materials, including two from Jilin City, two from Yanbian Prefecture, eleven from Baishan City, five from Huanren County, and one from Tonghua City. Subgroup III contained seventy-three materials, including eleven from Jilin City, fourteen from Yanbian Prefecture, twenty-one from Baishan City, three from Dandong City, seven from Huanren County, and seventeen from Tonghua City.

### 3.5. Genetic Differentiation Analysis of A. arguta Populations

The population differentiation coefficient (*Fst*) is an indicator of the gene frequency of the population. *Fst* is employed to measure the degree of genetic differentiation between different populations. *Fst* was 0.0249, and the population gene flow (*Nm*) was 9.7937 (Table 5). *Fst* was less than 0.05, and *Nm* was greater than 1, indicating that the level of gene exchange between populations of *A. arguta* was high and caused a higher genetic diversity.

The variance component ratios within and between the populations of *A. arguta* were 95% and 5%, respectively (Table 6). This shows that there is high genetic diversity within the populations of *A. arguta*. This may be attributed to the high gene flow between these individuals.

## 4. Discussion

Phenotypic diversity is an important part of genetic diversity and involves the phenotypic variation and distribution pattern of each population under different habitat conditions [34]. Phenotypic traits are the result of the interaction between the genotype and environment. Phenotypic trait variation is the manifestation of plant genetic diversity at the morphological level. The evaluation of the genetic diversity of phenotypic traits can determine the richness and variation in phenotypic traits as a whole, as well as explain the evolutionary patterns and evolutionary processes of species and populations [35]. In this study, the leaf quality traits of *A. arguta* germplasm resources in the Changbai Mountain area mainly exhibited abundant genetic variation in three areas: leaf shape, leaf base shape, and leaf color. The evaluation of leaf genetic diversity is of great significance for the germplasm innovation and variety breeding of *A. arguta*.

Cluster analysis of the leaf quality traits of *A. arguta* revealed that, apart from TH, the classification results of the populations were closely related to the geographical distance distribution. This agrees with the UPGMA cluster analysis. A close genetic relationship was observed between TH and the other populations. The large difference in leaf phenotype may be attributed to the difference in the unique geographical environment and climatic conditions of the TH population. In this study, the geographical and genetic relationships among the populations of *A. arguta* exhibited a significant positive correlation; that is, the farther the geographical distance, the farther the genetic relationship. This may be related to the effects of geographical isolation and environmental isolation, namely, the farther the physical distance, the greater the genetic difference, with heterogeneous environmental factors such as temperature, rainfall, and altitude exerting an effect on the genetic composition of the population [36,37,38].

The SSR genetic diversity analysis of the six populations of *A. arguta* indicated that there was a level of gene exchange and infiltration among the populations of *A. arguta*, and the genetic diversity among *A. arguta* is abundant. The population structure of six wild *A. arguta* natural populations and 148 individuals were divided into three subgroups. However, the cluster analysis of the six populations showed that the genetic distance between the populations was relatively small. This may be attributed to the small geographical span of the *A. arguta* used in this study, with just the Changbai Mountain area included in the sample, resulting in a small genetic distance among *A. arguta* populations. Lai et al. [17] collected and analyzed five populations of *A. arguta* in Shaanxi, Hunan, Sichuan, and Jilin, revealing that the large geographical span induced high genetic differentiation among populations. Hu et al. [39] speculated the presence of extensive refugees in the northeastern region, rather than smaller scattered refugee groups, during the last glacial period, resulting in extensive gene exchange and high genetic diversity within the *A. arguta* population. The *A. arguta* resources in the Qinling Mountains and Daba Mountains have high genetic diversity, and a high ploidy level can aid in improving the genetic diversity of species [16,18]. However, the *A. arguta* population in the Changbai Mountain area has a tetraploid ploidy level [40]. We speculated that there is only some scattered distribution of other ploidy. Wild *A. arguta* mainly pollinates via insect vectors [41]. The fruit is collected, carried, and eaten by humans and other animals. The fruit drop spreads with the stream, which eventually leads to the mixing of fruits and seeds of *A. arguta* from different populations. Gene flow results in a relatively uniform gene frequency distribution in each population. This also explains why individuals in each population are geographically clustered.

The Changbai Mountain area is one of the most widely distributed areas of wild *A. arguta* resources [42]. In recent years, with the uncontrolled logging and predatory resource utilization of human beings, the habitat of wild *A. arguta* has gradually decreased, and some populations have even become extinct. The harsh changes in global climatic conditions have also brought great challenges to the wild *A. arguta* population. Studies have shown that climate change is the main environmental factor affecting species distribution [43]. Severe climate change will change the suitable living area of plants and affect the growth, development, and yield of crops [44,45,46,47]. These phenomena have led to the current endangered state of wild *A. arguta*, which is in urgent need of protection [48]. Extensive genetic diversity evaluation is the basis for the protection and development of *A. arguta*. Based on these results, the following measures are suggested for the protection and maintenance of *A. arguta*: (1) the ex situ conservation of resources with unique traits, such as the establishment of a resource nursery to preserve the existing genetic resources; (2) in situ conservation of well-preserved populations, such as the establishment of nature reserves, to reduce resource loss and genetic diversity reduction; (3) hybrid breeding can be carried out for the resources with distant genetic relationships to create new germplasm; and (4) excellent resources can be selected by combining phenotypic traits and molecular markers. In summary, the study of the genetic diversity of *A. arguta* wild resources can provide a strong theoretical basis for the management, protection, and utilization of germplasm resources.

## 5. Conclusions

In this study, we found that 95% of the genetic variation came from within populations and 5% of the genetic variation came from among populations. Cluster analysis of leaf phenotypic quality traits of *A. arguta* germplasm was carried out by the group connection method, and the germplasm was divided into five groups. According to the genetic distance, the germplasm was divided into four groups by the UPGMA method. Population structure analysis divided 148 individuals into three subgroups. This study showed that the population of *A. arguta* in Changbai Mountain had large genetic variation and high genetic diversity. This finding will broaden the genetic basis of breeding germplasm of *A. arguta*, which is conducive to future research on the genetic improvement, forest resource management, protection, and breeding strategies of *A. arguta.*

## Figures and Tables

**Table 1:** Germplasm information of 6 populations of *A. arguta*.

Population	Sample Size	Latitude Range (°)	Longitude Range (°)	Altitude (m)
JL	15	43.08859–44.02439 N	125.80929–127.40944 E	230–305
YB	42	42.55463–43.33359 N	128.30504–129.68939 E	393–781
BS	55	41.56697–42.58434 N	127.04602–127.94427 E	522–095
HR	12	41.22214–41.27957 N	125.22703–125.28025 E	538–672
DD	5	40.69727–40.77578 N	124.74775–124.99916 E	343–499
TH	19	41.13954–42.28737 N	125.78193–126.15199 E	187–562
Total	148			

JL, YB, BS, TH, HR, and DD represent the populations of Jilin City, Yanbian City, Baishan City, and Tonghua City in Jilin Province and Huanren County and Dandong City in Liaoning Province, respectively.

**Table 2:** Six phenotypic quality traits and assignment criteria of the leaves of *A. arguta* germplasm resources.

Character Index	Assignment
Leaf shape	1 = Ovoid form; 2 = Broad oval; 3 = heart shape; 4 = oval
Tip shape	1 = tail tip; 2 = Sharp; 3 = Taper
Leaf margin	1 = sawtooth; 2 = Undulation
Leaf margin serration	1 = Thin single serrations; 2 = Coarse single sawtooth; 3 = Two-out complex serration
Leaf base shape	1 = Roundness; 2 = heart shape; 3 = wedge; 4 = Lopped section
Leaf color	1 = Aqua; 2 = green; 3 = Bottle green

**Table 3:** Distribution frequency and genetic diversity index of six phenotypic quality traits in leaves.

Phenotypic Quality Traits	Distribution Frequency				Genetic Diversity Index
	1	2	3	4	
Leaf shape	47.62%	20%	8.57%	23.81%	1.23
Tip shape	63.81%	19.05%	17.14%		0.91
Leaf margin	97.14%	2.86%			0.13
Leaf margin serration	59.80%	36.27%	3.92%		0.80
Leaf base shape	19.05%	31.43%	24.76%	24.76%	1.21
Leaf color	15.24%	37.14%	47.62%		1.01

Leaf shape: 1 = Ovoid form, 2 = Broad oval, 3 = heart shape, 4 = Oval; Tip shape: 1 = tail tip, 2 = Sharp, 3 = Taper; Leaf margin: 1 = sawtooth, 2 = Undulation; Leaf margin serration: 1 = Thin single serrations, 2 = Coarse single sawtooth, 3 = Two-out complex serration, Leaf base shape: 1 = Roundness, 2 = heart shape, 3 = wedge, 4 = Lopped section; Leaf color: 1 = Aqua, 2 = green, 3 = Bottle green.

**Table 4:** Genetic diversity of six populations by SSR analysis.

Population	Polymorphic Band Ratio (%)	*Na*	*Ne*	*I*	Ho	He
JL	95.45	1.955	1.671	0.554	0.392	0.379
YB	100.00	2.000	1.746	0.604	0.421	0.417
BS	100.00	2.000	1.708	0.589	0.407	0.403
HR	93.18	1.886	1.765	0.592	0.433	0.415
DD	84.09	1.795	1.672	0.521	0.404	0.363
TH	95.45	1.955	1.678	0.563	0.396	0.385
Mean	94.70	1.932	1.707	0.571	0.409	0.394

JL, YB, BS, TH, HR, and DD represent the populations of Jilin City, Yanbian City, Baishan City, and Tonghua City in Jilin Province and Huanren County and Dandong City in Liaoning Province, respectively. *Na*: observed alleles, *Ne*: effective alleles, *I*: Shannon’s information index, Ho: observed heterozygosity, He: expected heterozygosity.

**Table 5:** Nei’s analysis of genetic diversity in populations.

*Fst*	*Nm*
0.0249	9.7937

*Fst*: population differentiation coefficient, *Nm*: gene flow.

**Table 6:** Analysis of molecular variance (AMOVA) within and among *A. arguta* populations.

Source of Variation	Df	Sum of Square	Mean Square	Variance Component	Percentage of Variation (%)
Among pops	5	97.91	19.582	0.459	5
With pops	142	1332.86	9.386	9.386	95
Total	147	1430.77		9.845	100

Df: degree of freedom.

## Data Availability

Data will be made available on request.

## Supplementary Materials

The following supporting information can be downloaded at https://www.mdpi.com/article/10.3390/cimb47030207/s1.
