# Supplementary material for: Genetic Diversity of a Wild Actinidia arguta Population in Changbai Mountain Determined by Simple Sequence Repeat Markers
Source: Curr Issues Mol Biol. 2025 Mar 19;47(3):207. doi: 10.3390/cimb47030207 (PMC11940878; doi:10.3390/cimb47030207)
Supplement: Supplementary file 1 [file cimb-47-00207-s001.zip › Supplementary Table S1 Investigation on leaf quality traits of 6 populations of A.arguta.pdf]

Supplementary Table S1 Investigation on leaf quality traits of 6 populations of *A.arguta*

Population	Code	Leaf shape	Tip shape	Leaf margin	Leaf margin serration	Leaf base shape	Leaf color
JL	JL-01	Oval	Tail tip	Sawtooth	Coarse single sawtooth	Roundness	Dark green
	JL-02	Heart shape	Tail tip	Sawtooth	Coarse single sawtooth	Heart shape	Green
	JL-03	Ovoid form	Taper	Undulation	-	Roundness	Green
	JL-04	Broad oval	Tail tip	Sawtooth	Thin single serrations	Wedge	Dark green
	JL-05	Broad oval	Tail tip	Undulation	-	Lopped section	Dark green
	JL-10	Broad oval	Tail tip	Sawtooth	Thin single serrations	Wedge	Green
	JL-11	Broad oval	Taper	Sawtooth	Coarse single sawtooth	Wedge	Dark green
	JL-12	Ovoid form	Taper	Sawtooth	Coarse single sawtooth	Wedge	Dark green
	JL-13	Ovoid form	Tail tip	Sawtooth	Thin single serrations	Wedge	Dark green
	JL-14	Ovoid form	Tail tip	Sawtooth	Coarse single sawtooth	Lopped section	Green
	JL-15	Ovoid form	Tail tip	Sawtooth	Thin single serrations	Heart shape	Green
YB	YB-01	Broad oval	Tail tip	Sawtooth	Thin single serrations	Roundness	Green
	YB-02	Oval	Tail tip	Sawtooth	Coarse single sawtooth	Wedge	Dark green
	YB-04	Ovoid form	Tail tip	Sawtooth	Thin single serrations	Lopped section	Dark green
	YB-05	Heart shape	Taper	Sawtooth	Coarse single sawtooth	Heart shape	Dark green
	YB-08	Oval	Tail tip	Sawtooth	Coarse single sawtooth	Wedge	Green
	YB-09	Oval	Tail tip	Sawtooth	Thin single serrations	Roundness	Dark green
	YB-10	Ovoid form	Tail tip	Sawtooth	Thin single serrations	Heart shape	Aqua
	YB-12	Ovoid form	Taper	Sawtooth	Thin single serrations	Lopped section	Green

	YB-13	Ovoid form	Tail tip	Sawtooth	Thin single serrations	Lopped section	Green
	YB-14	Ovoid form	Sharp	Sawtooth	Thin single serrations	Lopped section	Aqua
	YB-15	Ovoid form	Tail tip	Sawtooth	Coarse single sawtooth	Heart shape	Aqua
	YB-16	Broad oval	Taper	Sawtooth	Thin single serrations	Lopped section	Green
	YB-18	Ovoid form	Tail tip	Sawtooth	Coarse single sawtooth	Lopped section	Dark green
	YB-19	Ovoid form	Tail tip	Sawtooth	Thin single serrations	Heart shape	Green
	YB-22	Oval	Tail tip	Sawtooth	Thin single serrations	Wedge	Green
	YB-25	Oval	Taper	Sawtooth	Coarse single sawtooth	Roundness	Green
	YB-30	Ovoid form	Tail tip	Sawtooth	Thin single serrations	Lopped section	Dark green
	YB-32	Ovoid form	Taper	Sawtooth	Thin single serrations	Heart shape	Dark green
	YB-35	Oval	Tail tip	Sawtooth	Thin single serrations	Heart shape	Green
	YB-36	Ovoid form	Tail tip	Sawtooth	Thin single serrations	Roundness	Aqua
	YB-37	Ovoid form	Taper	Sawtooth	Thin single serrations	Roundness	Dark green
	YB-39	Oval	Tail tip	Sawtooth	Thin single serrations	Lopped section	Green
	YB-40	Ovoid form	Tail tip	Sawtooth	Thin single serrations	Heart shape	Dark green
	YB-41	Ovoid form	Sharp	Sawtooth	Thin single serrations	Wedge	Dark green
	YB-43	Ovoid form	Taper	Sawtooth	Coarse single sawtooth	Wedge	Dark green
	YB-46	Ovoid form	Tail tip	Sawtooth	Thin single serrations	Roundness	Dark green
	YB-48	Ovoid form	Sharp	Undulation	-	Wedge	Aqua
	YB-49	Ovoid form	Taper	Sawtooth	Thin single serrations	Lopped section	Dark green
BS	BS-01	Ovoid form	Tail tip	Sawtooth	Two-out complex serration	Lopped section	Aqua

BS-02	Broad oval	Sharp	Sawtooth	Coarse single sawtooth	Heart shape	Dark green
BS-04	Heart shape	Sharp	Sawtooth	Coarse single sawtooth	Heart shape	Aqua
BS-05	Broad oval	Tail tip	Sawtooth	Thin single serrations	Heart shape	Dark green
BS-06	Broad oval	Tail tip	Sawtooth	Coarse single sawtooth	Heart shape	Dark green
BS-08	Ovoid form	Tail tip	Sawtooth	Thin single serrations	Heart shape	Dark green
BS-09	Broad oval	Tail tip	Sawtooth	Thin single serrations	Heart shape	Dark green
BS-10	Oval	Sharp	Sawtooth	Thin single serrations	Heart shape	Dark green
BS-11	Oval	Tail tip	Sawtooth	Thin single serrations	Lopped section	Green
BS-12	Ovoid form	Sharp	Sawtooth	Coarse single sawtooth	Heart shape	Green
BS-14	Ovoid form	Tail tip	Sawtooth	Thin single serrations	Roundness	Aqua
BS-15	Broad oval	Tail tip	Sawtooth	Thin single serrations	Heart shape	Aqua
BS-17	Ovoid form	Tail tip	Sawtooth	Coarse single sawtooth	Lopped section	Dark green
BS-19	Ovoid form	Tail tip	Sawtooth	Coarse single sawtooth	Heart shape	Green
BS-20	Oval	Tail tip	Sawtooth	Two-out complex serration	Roundness	Green
BS-21	Oval	Tail tip	Sawtooth	Coarse single sawtooth	Roundness	Dark green
BS-22	Oval	Sharp	Sawtooth	Coarse single sawtooth	Wedge	Dark green
BS-23	Oval	Tail tip	Sawtooth	Coarse single sawtooth	Lopped section	Dark green
BS-24	Ovoid form	Tail tip	Sawtooth	Coarse single sawtooth	Heart shape	Aqua
BS-25	Ovoid form	Tail tip	Sawtooth	Thin single serrations	Heart shape	Green
BS-27	Broad oval	Tail tip	Sawtooth	Coarse single sawtooth	Heart shape	Aqua
BS-28	Ovoid form	Tail tip	Sawtooth	Thin single serrations	Roundness	Dark green

	BS-29	Ovoid form	Sharp	Sawtooth	Thin single serrations	Heart shape	Dark green
	BS-30	Ovoid form	Tail tip	Sawtooth	Thin single serrations	Heart shape	Dark green
	BS-31	Ovoid form	Tail tip	Sawtooth	Coarse single sawtooth	Roundness	Green
	BS-32	Ovoid form	Tail tip	Sawtooth	Coarse single sawtooth	Wedge	Green
	BS-33	Broad oval	Tail tip	Sawtooth	Thin single serrations	Roundness	Green
	BS-35	Ovoid form	Tail tip	Sawtooth	Two-out complex serration	Heart shape	Green
	BS-36	Ovoid form	Tail tip	Sawtooth	Thin single serrations	Lopped section	Green
	BS-38	Broad oval	Tail tip	Sawtooth	Thin single serrations	Roundness	Green
	BS-39	Ovoid form	Tail tip	Sawtooth	Coarse single sawtooth	Wedge	Green
	BS-42	Broad oval	Taper	Sawtooth	Thin single serrations	Lopped section	Green
	BS-48	Oval	Tail tip	Sawtooth	Thin single serrations	Wedge	Dark green
	BS-49	Oval	Sharp	Sawtooth	Thin single serrations	Wedge	Green
	HR-01	Ovoid form	Tail tip	Sawtooth	Thin single serrations	Lopped section	Aqua
	HR-02	Ovoid form	Tail tip	Sawtooth	Thin single serrations	Wedge	Green
	HR-03	Heart shape	Tail tip	Sawtooth	Thin single serrations	Lopped section	Green
	HR-04	Heart shape	Taper	Sawtooth	Thin single serrations	Lopped section	Green
HR	HR-05	Ovoid form	Tail tip	Sawtooth	Thin single serrations	Wedge	Green
	HR-06	Oval	Tail tip	Sawtooth	Thin single serrations	Wedge	Aqua
	HR-07	Oval	Sharp	Sawtooth	Coarse single sawtooth	Lopped section	Green
	HR-08	Broad oval	Sharp	Sawtooth	Thin single serrations	Roundness	Green
	HR-09	Ovoid form	Sharp	Sawtooth	Thin single serrations	Wedge	Dark green

	HR-10	Ovoid form	Sharp	Sawtooth	Thin single serrations	Wedge	Green
	HR-11	Oval	Sharp	Sawtooth	Coarse single sawtooth	Wedge	Aqua
	HR-12	Oval	Tail tip	Sawtooth	Coarse single sawtooth	Wedge	Green
DD	DD-01	Heart shape	Taper	Sawtooth	Two-out complex serration	Heart shape	Green
	DD-02	Ovoid form	Taper	Sawtooth	Coarse single sawtooth	Lopped section	Dark green
	DD-03	Broad oval	Tail tip	Sawtooth	Thin single serrations	Lopped section	Dark green
	DD-04	Broad oval	Sharp	Sawtooth	Thin single serrations	Heart shape	Dark green
	DD-05	Broad oval	Sharp	Sawtooth	Thin single serrations	Lopped section	Green
TH	TH-02	Heart shape	Tail tip	Sawtooth	Coarse single sawtooth	Heart shape	Dark green
	TH-03	Oval	Tail tip	Sawtooth	Coarse single sawtooth	Wedge	Dark green
	TH-06	Oval	Tail tip	Sawtooth	Coarse single sawtooth	Roundness	Aqua
	TH-07	Ovoid form	Tail tip	Sawtooth	Coarse single sawtooth	Heart shape	Aqua
	TH-08	Ovoid form	Tail tip	Sawtooth	Thin single serrations	Roundness	Dark green
	TH-11	Heart shape	Taper	Sawtooth	Coarse single sawtooth	Heart shape	Dark green
	TH-16	Heart shape	Tail tip	Sawtooth	Thin single serrations	Heart shape	Dark green
	TH-17	Broad oval	Taper	Sawtooth	Thin single serrations	Heart shape	Dark green
	TH-18	Ovoid form	Sharp	Sawtooth	Coarse single sawtooth	Wedge	Green
	TH-19	Oval	Sharp	Sawtooth	Thin single serrations	Roundness	Green
	TH-20	Oval	Tail tip	Sawtooth	Thin single serrations	Wedge	Dark green
