# Supplementary material for: Genetic Diversity of a Wild Actinidia arguta Population in Changbai Mountain Determined by Simple Sequence Repeat Markers
Source: Curr Issues Mol Biol. 2025 Mar 19;47(3):207. doi: 10.3390/cimb47030207 (PMC11940878; doi:10.3390/cimb47030207)
Supplement: Supplementary file 1 [file cimb-47-00207-s001.zip › Supplementary Table S2 Names and sequence information of 31 pairs of SSR primers.pdf]

Supplementary Table S2 Names and sequence information of 31 pairs of SSR primers

Primer	Primer sequence (5'-3')	Allele size (bp)	Optimum annealing temperature (℃)
Arg-001	F：CTGAGGTGACAGCGGCGGATAC<br>R：CGGGCTTTTGAACCTCGGAAAC	130-150	60
Arg-004	F：GCAAACTGGCAGCGCAAAA<br>R：TTGGAGCTCGCGGTTGGAATAC	250-260	60
Arg-012	F：CACCGACACGAGTACTGCTTCT<br>R：CCCTGGCAACCACAACGTACTT	120-136	60
Arg-013	F：CCAGTGCCAAAGACTGCATTGA<br>R：AACCCGTCAGCCTATCCCTAGA	130-140	61
Arg-019	F：CTCCTGGACGCATGCTGATAGA<br>R：CGTCAACGCCAAGCTTCTCT	170-186	61
Arg-022	F：AGGACTCCAACAACTTCAAGGT<br>R：TGCACCCACAATCCCTGAATAT	210-225	61
Arg-025	F：CCCACCACCGCCTTCTCCTTCA<br>R：ACCTCCACAACAGCGCCTTGAA	153-180	60
Arg-028	F：TGGGTGAGGAGCTGTCGGATGT<br>R：GAAAATCAACACCAATGCTGTT	175-188	61.5
Arg-034	F：TTGGTGGTGGTGCTTGCTTCTT<br>R：TCATCCTTCTCCTCGTCCTTTT	205-213	60
Arg-040	F：GGAGTACTGTGCATGGCCTTCT<br>R：AGTGGTACGTTGCTCTGATATT	187-206	59.5
Arg-041	F：CCCTTTGGTGCTCTATTGATTA<br>R：TCACGGTGACTGCTTTCTCCTC	150-164	60
Arg-055	F：CCGGATAGGCACCCATCGAATA	135-160	60

	R：ACCCCTCACTCTCTCCCACGA		
Arg-057	F：GCAGACGAGACTTGCCAAGGAA<br>R：TGGGTTGCCTGGTCCTCAATTT	145-182	60
Arg-062	F：GGGAGGTGTAGGCAAAGGAATT<br>R：TCAATCCCCACCACCGCTAGCA	138-154	60
Arg-064	F：ACTTTGCACAACCGGTCATAAA<br>R：CATGGTGATGAGGCATGAATAG	172-193	61
Arg-068	F：GGAAAGGTGGTGGCGGTGGATT<br>R：CGCCCGGTCCCTCACAAAAC	175-190	60
Arg-074	F：CGGTGGTAGACGTGGTGGTGAT<br>R：CGAAAGCCCAAAACCCGTGCAT	225-240	59
Arg-075	F：CACCGCCCCGATCTCCTCCT<br>R：GCTCCAGCGGCAACATAAGC	145-166	60
Arg-076	F：AAGCCGCAAAGAGGGAGGAGG<br>R：GCTTCCTGTTGCCTGGCCTCTT	185-194	58
Arg-087	F：GGGTCCTGCGCCCACTATC<br>R：AGTGGAGGTGGTGGCGGAAGAG	156-180	61
Arg-091	F：AGCGCACCTCTCTCCCCTAATC<br>R：AGCTCGGCGTTGTTGGGGTCCA	170-220	61
Arg-092	F：CCGGGAAGCGGATGGGGTTATG<br>R：TCCTTGGAGACCGATGGCAAAC	196-235	60
Arg-093	F：TCAGCCGCCACGAACTACAACA<br>R：GGCCCCGAGTCCCATTCCAAAA	141-160	60
Arg-112	F：GCAATGGAAGGCTGTGATCC<br>R：CCTCGTAGTCCAAGCTCGAA	175-189	60

Arg-126	F：CCTAAGCAACCGTGATGTGG<br>R：CCCCTTTGTCGTCGCTACTA	170-185	60
Arg-128	F：CAGCTTCCAAATACTTCGGCA<br>R：GTGACTATAGGCGACGTGGT	163-180	60
Arg-129	F：ACACCTACCACGTCGTTTCT<br>R：ATCATTCCGAACCAGGCCTT	135-170	61
Arg-131	F：GCGGAGAGGTAGGAATGGAG<br>R：CACCAACGACTCCTTCCTCT	162-175	60
Arg-134	F：CTTTAGTCCAGCGCAACCTC<br>R：CCGTTTCAAGTGAGGTGACC	162-180	59.5
Arg-136	F：TGTGTTCATTGGTGCTTGCC<br>R：AACACAAGACCACCACTCCA	165-210	60
Arg-139	F：CCAACAGAGAGCCCAAAACC<br>R：GAGATCGTCTAGGAGTCGCC	132-165	60.5
